# Transcriptomic Analysis of Human Polarized Macrophages: More than One Role of Alternative Activation?

**DOI:** 10.1371/journal.pone.0119751

**Published:** 2015-03-23

**Authors:** Eleonora Derlindati, Alessandra Dei Cas, Barbara Montanini, Valentina Spigoni, Valentina Curella, Raffaella Aldigeri, Diego Ardigò, Ivana Zavaroni, Riccardo C. Bonadonna

**Affiliations:** 1 Unit of Diabetes and Metabolism, Department of Clinical and Experimental Medicine, University of Parma and AOUI of Parma, Parma, Italy; 2 Division of Endocrinology, Department of Clinical and Experimental Medicine, University of Parma and AOUI of Parma, Parma, Italy; 3 Laboratory of Functional Genomics and Protein Engineering, Biochemistry and Molecular Biology Unit, Department of Life Sciences, University of Parma, Parma, Italy; University of Catanzaro Magna Graecia, ITALY

## Abstract

**Background:**

Macrophages are a heterogeneous cell population which in response to the cytokine milieu polarize in either classically activated macrophages (M1) or alternatively activated macrophages (M2). This plasticity makes macrophages essential in regulating inflammation, immune response and tissue remodeling and a novel therapeutic target in inflammatory diseases such as atherosclerosis. The aim of the study was to describe the transcriptomic profiles of differently polarized human macrophages to generate new hypotheses on the biological function of the different macrophage subtypes.

**Methods and Results:**

Polarization of circulating monocytes/macrophages of blood donors was induced *in vitro* by IFN-γ and LPS (M1), by IL-4 (M2a), and by IL-10 (M2c). Unstimulated cells (RM) served as time controls. Gene expression profile of M1, M2a, M2c and RM was assessed at 6, 12 and 24h after polarization with Whole Human Genome Agilent Microarray technique. When compared to RM, M1 significantly upregulated pathways involved in immunity and inflammation, whereas M2a did the opposite. Conversely, decreased and increased expression of mitochondrial metabolism, consistent with insulin resistant and insulin sensitive patterns, was seen in M1 and M2a, respectively. The time sequence in the expression of some pathways appeared to have some specific bearing on M1 function. Finally, canonical and non-canonical Wnt genes and gene groups, promoting inflammation and tissue remodeling, were upregulated in M2a compared to RM.

**Conclusion:**

Our data in *in vitro* polarized human macrophages: 1. confirm and extend known inflammatory and anti-inflammatory gene expression patterns; 2. demonstrate changes in mitochondrial metabolism associated to insulin resistance and insulin sensitivity in M1 and M2a, respectively; 3. highlight the potential relevance of gene expression timing in M1 function; 4. unveil enhanced expression of Wnt pathways in M2a suggesting a potential dual (pro-inflammatory and anti-inflammatory) role of M2a in inflammatory diseases.

## Background

Macrophages (Mϕ) are implicated in the pathophysiology of different clinical conditions, including infectious [1], inflammatory and autoimmune diseases, atherosclerosis [2] and tumor progression [3]. In atherogenesis, Mϕ avidly take up lipoproteins, subsequently transformed into foam cells, which are involved in plaque progression, and release pro-inflammatory mediators, which are implicated in the pathogenesis of vulnerable/complicated plaques. However, Mϕ are a heterogeneous cell population, which in response to cytokines [4], released by activated lymphocytes [5] or damaged tissues, differentiate in either classically activated Mϕ (M1) or alternatively activated Mϕ (M2) [6, 7]. Although Mϕ activation *in vivo* is a continuum of functional phenotypes with intermediate or overlapping features, in *in vitro* studies these extreme Mϕ phenotypes (M1-M2) are commonly used.

M1 Mϕ result from the classical activation pathway triggered by inflammatory mediators such as IFN-γ (interferon gamma) and IL (interleukin)-1β, alone or in concert with microbial stimuli, like LPS (lipopolysaccharide) [8]. M1 Mϕ in turn release pro-inflammatory cytokines such as IL-6, IL-12 and TNF-α (Tumor Necrosis Factor alpha), and are characterized by the-expression of MHC (Major Histocompatibility Complex) class II molecules and by the capability of antigen presentation [9].

Conversely, M2 Mϕ result from exposure of signals other than IFN-γ and LPS, released from Th2 [10] and Treg cells [11], and express high levels of mannose and scavenger receptor CD163 [12]. The present standardized classification of cultured M2 holds that M2a are obtained after exposure to IL-4/IL-13; M2b after exposure to immune-complex and M2c (deactivated Mϕ) after exposure to IL-10.

M2a Mϕ are involved in immunoregulation, tissue repair [13] and tumor progression [3], but they are not efficient in antigen presentation and microbial killing, nor produce pro-inflammatory cytokines.

M2c Mϕ are characterized by the secretion of IL-10 and TGF-β (Transforming Growth Factor beta), the suppression of the expression of inflammatory cytokines, and by the lack of any ability to kill pathogens. Accordingly, M2c Mϕ promote no inflammatory or immune response and show enhanced phagocytic activity [14]. Further Mϕ phenotypes include Mox, Mhem and M4, induced by oxidized lipids, hemoglobin and platelet factor 4, respectively [15, 16].

Thus, as a consequence of different environmental signals, Mϕ can undergo different polarizations and play diverse, even opposite, roles in the pathogenesis of many conditions and diseases.

Mϕ, therefore, are novel, but complex, cellular targets in the treatment of inflammatory diseases, including atherosclerosis.

Gene expression analysis during the polarization process may be of help in identifying novel targets with a pivotal role in the regulation of Mϕ phenotype. Several studies have investigated the transcription profile of differently polarized Mϕ [6, 17–19]. However, in those studies only a single time point of each Mϕ phenotype was analyzed and gene expression profiles were compared between different subphenotypes and not to a time control of resting Mϕ. Thus, on one side, the dynamic phase of Mϕ polarization could not be studied, and, on the other side, the extent of changes in gene expression profiles induced by the polarization process may not have been properly estimated. Both notions may add significantly to our understanding of Mϕ biology and role in a number of inflammatory conditions.

The aim of this study was to assess the time course of the transcriptomic platforms during *in vitro* polarization of human Mϕ to M1 or M2a or M2c phenotype. We argued that close scrutiny of their complete gene expression “fingerprints” could generate new hypotheses on the biology of different Mϕ subtypes.

## Methods

### Ethics Statement

Buffy coats from three healthy donors were obtained according to protocols reviewed and approved by the Ethical Committee of University of Parma. The Ethical Committee waived the need for consent because the biologic specimens were fully anonymized.

### Cell preparation

Peripheral Blood Mononuclear Cells (PBMCs) were isolated from three healthy donor buffy coats by Ficoll density gradient centrifugation (Lymphoprep, Sentinel Diagnostic, Milan, Italy) and monocytes were obtained using Dynabeads negative isolation kit (Invitrogen, Carlsbad, CA, USA), following manufacturer’s instructions. Purity was assessed by flow cytometry using anti-CD45 and anti-CD14 antibodies and was routinely greater than 85%. A total of 10^6^ cells/well were seeded in uncoated 24-well plate in RPMI 1640 medium (Gibco, Invitrogen, Carlsbad, CA, USA) supplemented with 10% FBS (fetal bovine serum), 1% L-glutamine, 1% pen/strep, 1% amphotericin B and 50ng/ml Macrophage-Colony Stimulating Factor (M-CSF) (Sigma Aldrich, St Louis, Missouri, USA) and cultured at 37°C and 5% CO_2_ as previously reported [20]. On day 6, the culture medium was replaced and stimuli were added. By medium replacement, lymphocytes were totally removed from wells. Classical activation (M1) was induced using LPS (100ng/ml) and IFN-γ (20ng/ml), alternative activation (M2a) was triggered with IL-4 (20ng/ml) whereas deactivation (M2c) was obtained with IL-10 (20ng/ml). LPS and all cytokines were purchased from Sigma Aldrich. Resting Mϕ (RM) were cultured without stimuli and used as control population. Cells were lysed at 6, 12, and 24 hours after the addition of different cues (or at equivalent culture time for RM).

### RNA isolation

Cells were harvested in 1ml of Qiazol (Qiagen, Valencia, CA, USA) and total RNA isolated according to the manufacturer’s instructions. Total RNA yield and the absence of DNA contamination were checked using a NanoDrop ND-1000 UV-Vis Spectrophotometer (NanoDrop Technologies, Wilmington, DE, USA). RNA quality was assessed measuring the 28S/18S rRNA and the RNA Integrity Number (RIN) with Bioanalyzer 2100, using RNA 6000 Nano Chips (Agilent Technologies, Santa Clara, CA, USA). RNA samples with RIN values > 8, 260/280 absorbance ratios >1.8 and 260/230 absorbance ratios >1.5 were considered suitable for microarray analysis.

### Gene expression array

A total of 500 ng of each RNA sample was reversely transcribed into cDNA, and subsequently amplified and labeled with Cy5 dye following Agilent’s Two-Color Microarray-Based Gene Expression Analysis protocol version 6.0. The same amount of RNA from a commercially-available pool of human leucocyte total RNA (Clontech, Mountain View, CA, USA) was reversely transcribed, amplified and Cy3-labeled to be used as reference. Spike-in RNA (Agilent Technologies) was used as internal control. RNA references and samples were labeled separately and then hybridized together on 4X44 Whole Human Genome Agilent Microarray slides (Agilent Technologies). Slides were scanned with an Agilent's G2565AA Microarray Scanner System. Dye-normalized, background-subtracted log-ratios of sample to reference expression were calculated using Agilent’s Feature Extraction Software version 9.5. Hybridization quality was checked using the software’s quality report.

### Real time PCR

To confirm Mϕ phenotype and to validate microarray findings, real time PCR (qPCR) analysis was performed on candidate genes. Four replicates of RNA samples were used for PCR analysis. cDNA was obtained using “High Capacity RNA-to-cDNA Kit” (Applied Biosystem, Life technologies, Foster City, California, USA), following manufacturer’s instruction. *IL-6*, *IL-8*, *PTGS2* (Prostaglandin-endoperoxide synthase 2), *SOCS1* (Suppressor of cytokine signaling1), *WNT5A (*wingless-type MMTV integration site family, member 5a), *SOD2* (superoxide dismutase 2), *ALOX15 (*Arachidonate 15-lipoxygenase*)*, *CD200R*, *CD163*, *WNT5b*, *GPD2* (mitochondrial Glyceraldehyde-3-phosphate dehydrogenase),*COX5a* (cytochrome c oxidase subunit 5a) and *CD206* gene expression was evaluated using TaqMan Gene expression Master Mix (Applied Biosystems) with TaqMan primers and probes on a StepOne Real-Time PCR system (Applied Biosystem). Thermal cycling conditions were as follows: 50°C for 2 min, 95°C for 10 min, followed by 40 amplification cycles (95°C for 15 s; 60°C for 1 min). Gene expression values were calculated based on the -ΔΔCt method [21]. The relative level of mRNA expression was calculated using as reference the geometric mean of human *GAPDH* (glyceraldehyde 3-phosphodehydrogenase), *ACTB* (β-actin), *RPS18* (ribosomal protein S18) and *YWHAZ* (Tyrosine 3-Monooxygenase/Tryptophan 5-Monooxygenase Activation Protein).

### Statistical analysis

Acquired array images were processed using the GeneSpring GX v11.5 software package (Agilent Technologies). Probes detectable in at least one condition (two out of the three biological replicates) were kept for further data analysis (27920 probes). A Hierarchical clustering was performed on an ANOVA (Benjamini Hochberg correction p-value < 0.05) filtered gene set, to highlight major differences among conditions. Differentially expressed genes were identified by Moderated t-test (Benjamini Hochberg correction p-value < 0.05, and Fold Change > = 2), between each condition and the corresponding time point in RM condition. Moderated t-test was performed to compare the transcriptome profile of RM at different time point (RM_6h vs RM_12h; RM_6h vs RM_24h; RM_12h vs RM_24h) This test was used as a first approach to assess whether there was any significant differentially expressed gene between one treatment condition and the corresponding RM time point. Further analyses were performed only in those comparisons which showed at least one differentially expressed gene (Gene Set Enrichment Analysis, GSEA), and in the only stimulus (M1) which resulted in at least one differentially expressed gene at all time points (STEM). GSEA analysis (http://www.broadinstitute.org/gsea) was performed on all detected probes to identify molecular pathways significantly over-represented among the upregulated and downregulated genes, and to compare the expression profiles with other published studies. In particular, data published by Martinez and colleagues [6] were retrieved from Gene Expression Omnibus (GSE5099) and compared with the present transcription profiles using GSEA. For this purpose, differentially expressed genes between “Macrophage at 7 days” condition and either “classical or M1 activated macrophages” or “Alternative or M2 activated macrophages” conditions (18 hours stimuli) were identified using the GEO2R tool, and the top 200 most upregulated or downregulated genes were kept to generate the M1_UP, M1_DN, M2a_UP, and M2a_DN gene sets. Enrichment maps issuing from GSEA were visualized using Cytoscape [22, 23].

The Short Time-series Expression Miner (STEM) suite was used to identify relevant GO terms associated with co-expressed genes in M1 treatment time series [24]. At this purpose, for each time point M1 expression data were normalized to the corresponding RM and log2 transformed, prior loading into STEM software. A zero time point was added to all expression profiles. The maximum number of model profiles was set to 20 and three unit changes in model profiles were allowed between time points. A default setting was left for the other options.

## Results

### Global analysis

**Fig 1:**
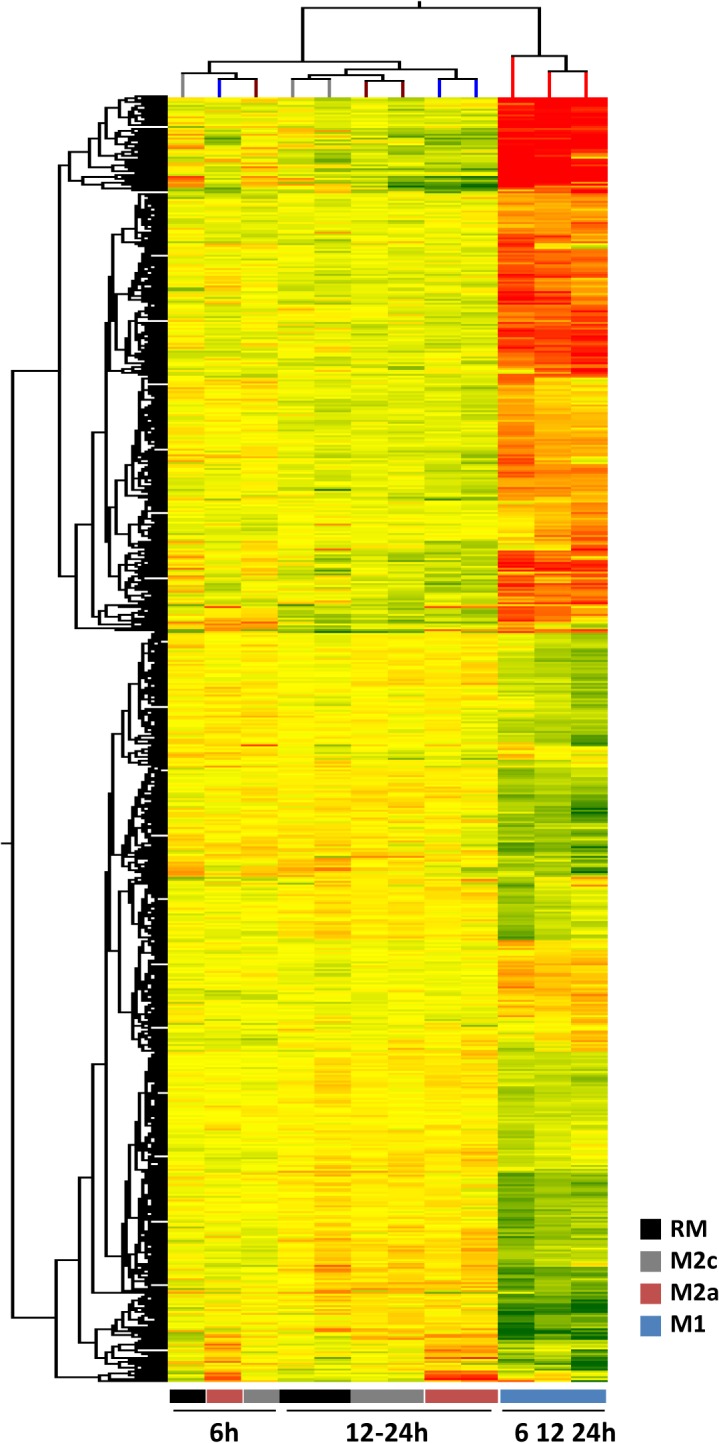
Hierarchical clustering HCL. HCL analysis among resting (RM), classical (M1), alternative (M2a) and deactivate Mϕ (M2c) in the three point of time course (6, 12 and 24 h) on an ANOVA-filtered gene set. The red, yellow and green colors represent higher than average, close to average and lower than average gene expression respectively. X axis reports the type of activated Mϕ while genes are listed in the y axis.

Hierarchical clustering (HCL) was performed on an ANOVA-filtered gene set as a first descriptive step to compare similarities in transcriptomic profile among the different experimental conditions and to visualize the relative contributions of stimulus type *vs* time course on gene expression profile (Fig. 1). HCL promptly separated transcriptomic profiles of M1 activation from all the others. Furthermore, within M1 the 6^th^ hour time point tended to be separated from both 12 and 24 hours time points, to suggest that a dynamic process had been captured. RM, M2a and M2c tended to cluster together, but within them, time appeared to be a better clustering factor than Mϕ subtype, in that the 6^th^ hour time point, as with M1, was separated from the other 2 time points. However, within the 12–24 hours interval, type (absence) of activation was a better discriminating factor than time (Fig. 1).

As suggested by the global HCL analysis, during polarization towards the M1 phenotype, hundreds to thousands genes were significantly modulated (Moderated t-test, BH correction p-value < 0.05) at all three time points, when compared to RM at the same time points (Table 1). A complete list of genes significantly modulated during activation of the M1 phenotype is available on S1 Table.

**Table 1:** Number of genes up- and down-regulated in polarized Mϕ at specific time points.

**Cell phenotype**	**Total analyzed genes**	**Up-regulated 6h**	**Up-regulated 12h**	**Up-regulated 24h**	**Down-regulated 6h**	**Down-regulated 12h**	**Down-regulated 24h**
M1	41000	1660	655	1692	988	144	2210
M2a	41000	0	15	19	0	2	11
M2c	41000	0	0	0	0	0	0

Significantly differently expressed probes(p <0.05 moderated t test with Benjamini Hochberg)

During polarization towards the M2a phenotype, about 50 genes were differentially expressed when compared to RM at the same time points (Table 1). A complete list of genes significantly modulated during activation of the M2a phenotype is available on S2 Table.

During polarization towards the M2c phenotype, no significant differences in individual gene expression were found at any time point, when compared to RM (Table 1).

To verify whether the transcriptome profile of resting Mϕ was stable throughout the different time points, we carried out within-time moderated t-tests (RM_6h vs RM_12h; RM_6h vs RM_24h; RM_12h vs RM_24h), but no significant differences in gene expression were found using the multiple testing correction filter (Benjamini Hochberg correction, p<0.05).

### M1 Mϕ activation

M1 Mϕ showed an increased expression of a host of genes involved in inflammation (e.g. *SOCS3* and *1*, *PTGS2*, *JAK*1, *NF-*kB1) and apoptosis (e.g. *FAS*, *CASP5* and *7*, interferon regulatory factor *IRF1*, *IRF5* and *IRF7*) at each time point.

In addition, several novel pro-inflammatory genes were also upregulated, such as *CD38*, involved in cell adhesion and phagocytosis [25], and *EREG2* (epiregulin2), involved in pro-inflammatory cytokine production, antigen presentation, and innate immunity [26]. The expression of several marker genes, which are typical of M1 polarization, was validated by qPCR (S1 Fig., panel A).

**Fig 2:**
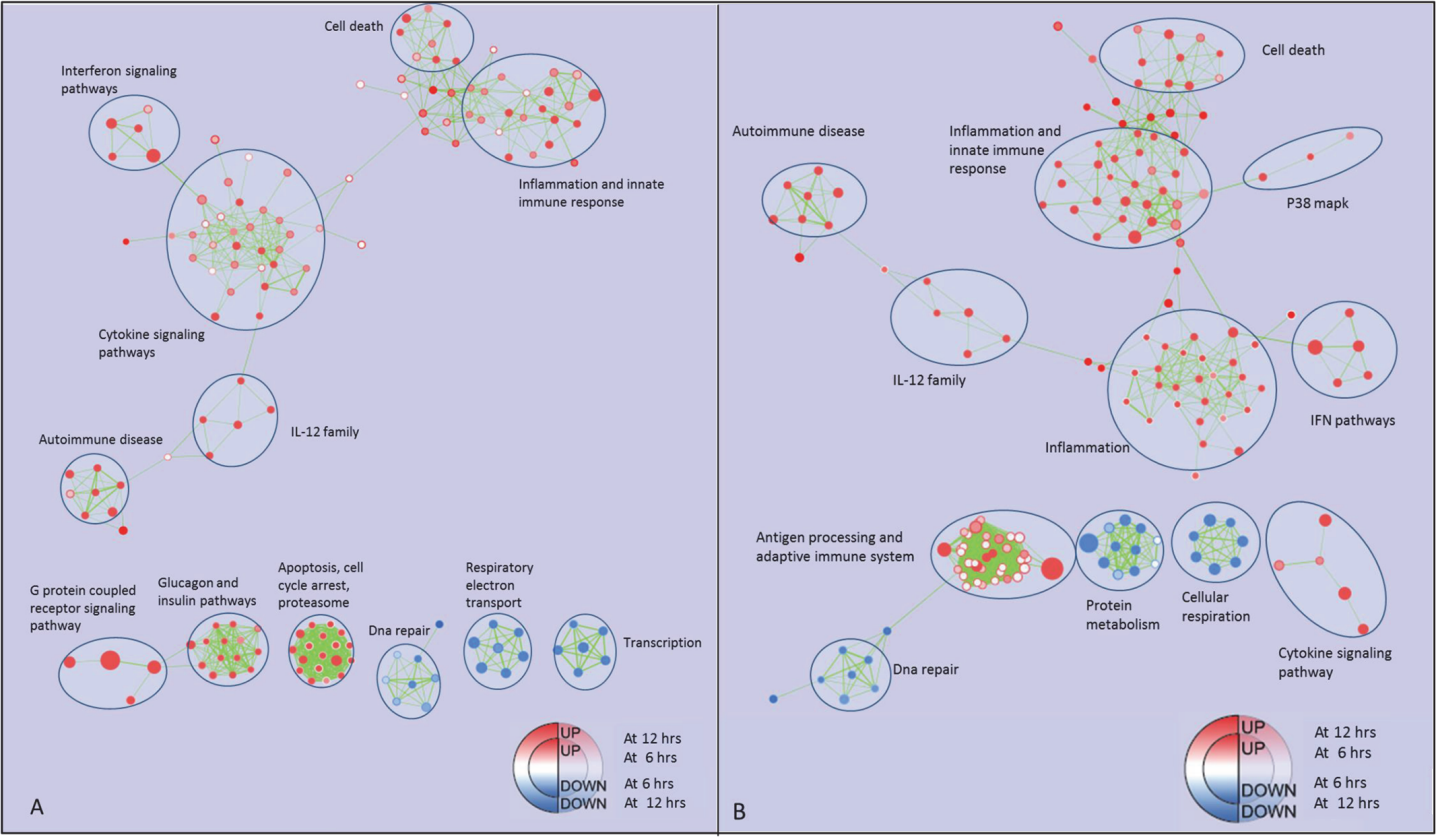
Enrichment map for M1 *vs* RM at 6, 12 and 24 hours. Panel A: the map displays the enriched pathways (p-value < 0.01, FDR < 20%), issued from GSEA, in M1 *vs* RM at 6 and 12 hours of culture. Enrichments were mapped to the inner node area and to the node borders, respectively. Panel B: the map displays the enriched pathways in M1 *vs* RM at 12 and 24 hours of culture. Enrichments were mapped to the inner node area and to the node borders, respectively. Red and blue dots represent up- and down-regulated gene-sets respectively, in M1 *vs*. RM. Edge thickness represents the fraction of common genes between pathways.

To gain more insight into the dynamics and biological meaning of gene expression during activation to the M1 phenotype, GSEA was performed and the GO terms and pathways which were significantly enriched (p-value <0.01, FDR<20%) in M1 *vs* RM at 6, 12 and 24 hours were identified. The results were visualized as networks (enrichment maps). Each enrichment map was generated by mapping a time point enrichment to the node center and the next time point enrichment to the node border (Fig. 2, panel A and B). IFN signaling and IL-12 family pathways were upregulated at each time point, whereas the pathways of cellular respiration were downregulated. Gene involved in cell death, p38 MAPK (*Mitogen-activated protein kinases)* pathway and inflammation enhanced their expression from 6 to 24 hours. Conversely, gene sets regarding DNA repair and mitochondrial metabolism decreased their expression over time. In spite of the increase of inflammation-related genes, the IL-10 signaling pathway underwent a significant upregulation from 6 hours to 24 hours.

**Fig 3:**
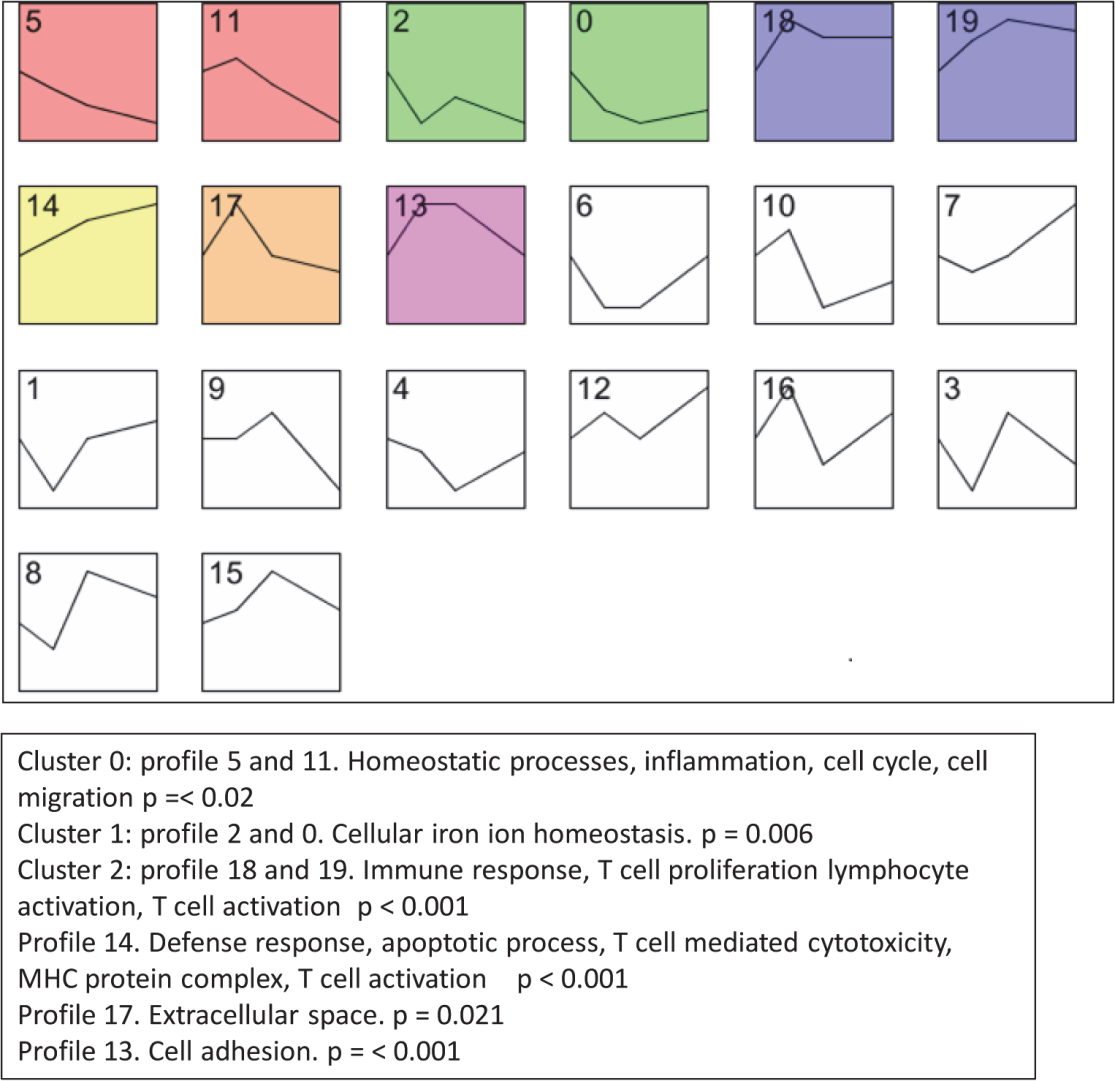
Time-Series Clustering of M1 Microarray Gene Set Data Using STEM. The expression data were loaded onto STEM platform and distinct temporal expression profiles were generated, which differentiate between real and random patterns. Profiles are numbered from 0 to 19. Each box corresponds to a model expression profile. Significant expression profiles are highlighted in color. Clusters with similar colors show similar patterns. To all expression profiles a zero time point was added to serve the control value. Genes are assigned to the most closely matching profile by statistical analysis. The X-axis represents hours after stimuli addition and the Y-axis denotes fold-increase or decrease in expression in log_2_ scale. Cluster were described with the main representative GO term.

Since a significant differential expression (M1 *vs* RM) was found at each time point, the time course of normalized Mϕ classical activation was analyzed by partitioning differentially expressed genes into 9 significantly represented expression profiles, grouped in 6 different clusters using STEM. This analysis aims at clarifying the timing of events during M1 polarization. The results are shown in Fig. 3. Cell adhesion pathways (profiles 13 and 17) showed an early peak of expression followed by a decline. Functional categories associated to innate immune response, lymphocyte activation and response to stress showed a robust and stable upregulation from 6 to 24 hours. Pathways related to apoptosis (profile 14) showed a linear increase in expression from 6 to 24 hours.

### M2a Mϕ activation

During M2a activation, there was a significant upregulation of *CCL26*, *ALOX15* and *IRF4*, as described previously [12, 27]. Moreover there was an overexpression of *WNT5b*, a gene found to be involved in adipogenesis and type 2 diabetes [28–30]. Since there were only few genes showing significant differences in expression when compared to RM, no time course analysis was performed. The expression of markers which characterized M2 polarization was validated by qPCR (S1 Fig., panel B).

**Fig 4:**
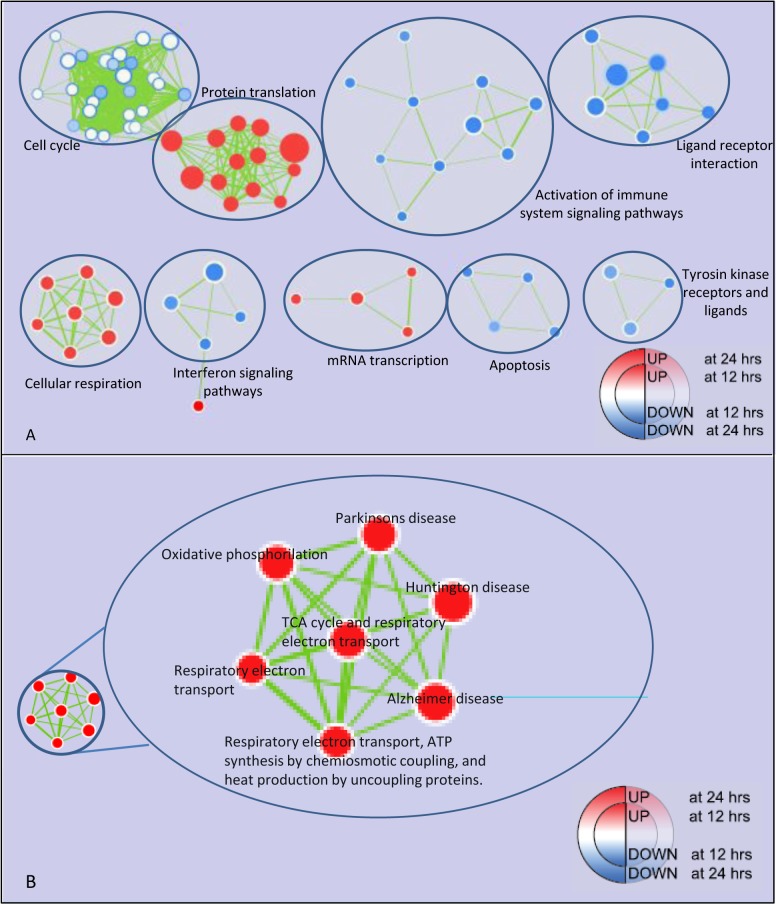
Enrichment map for M2a *vs* RM at 12 and 24 hours. Panel A: the map displays the enriched pathways (p-value < 0.01, FDR < 20%), issued from GSEA, in M2a *vs* RM at 12 and 24 hours of culture. Enrichments were mapped to the inner node area and to the node borders, respectively. Red and Blue dots represent up- and down-regulated gene-sets respectively, in M2a *vs* RM. Edge thickness represents the fraction of common genes between pathways. Panel B: zoom in the cellular respiration cluster.

**Fig 5:**
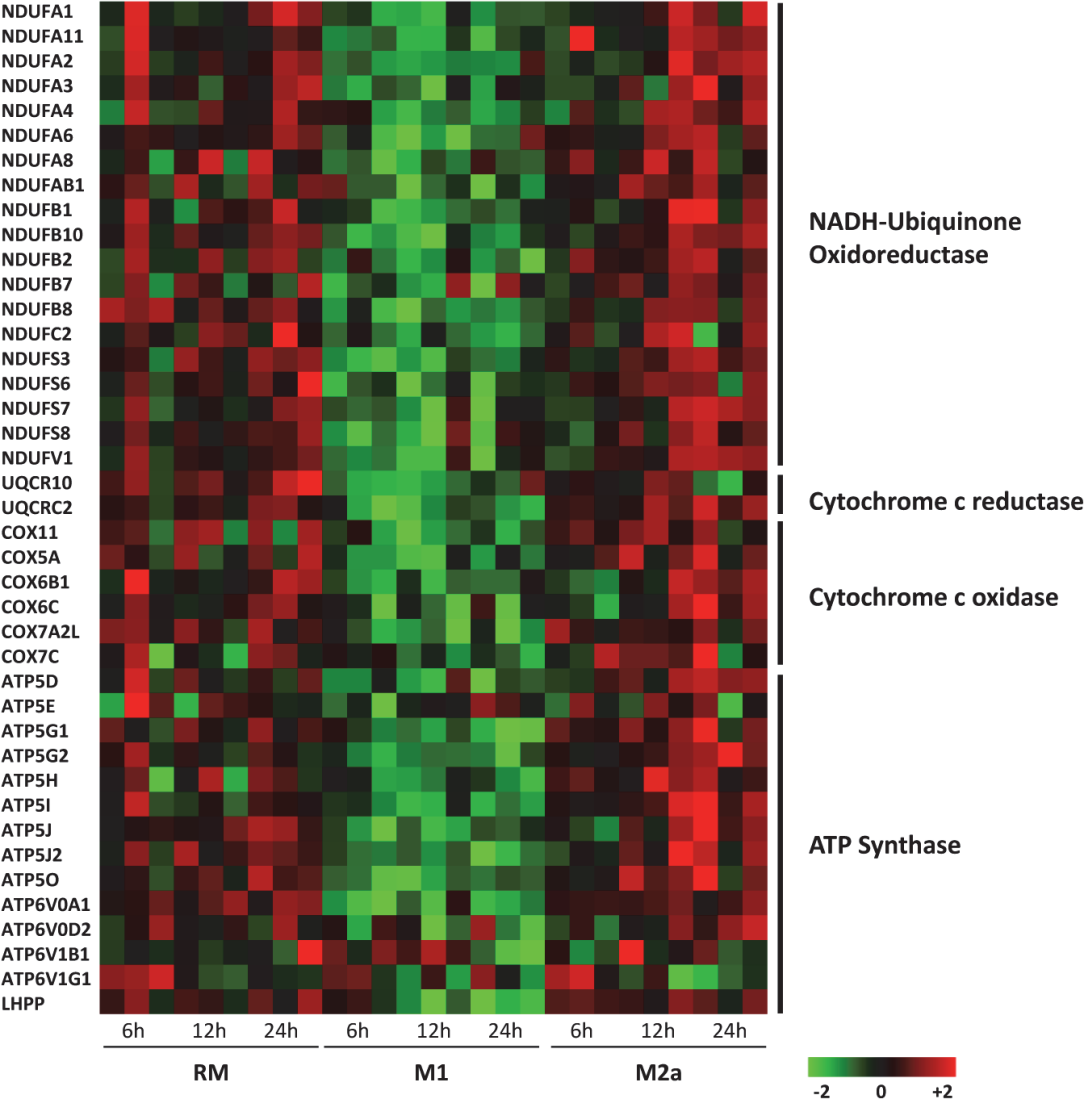
Heat-map displaying gene expression profiles of Oxidative phosphorylation. The heat map represents the mRNA expression of Oxidative Phosphorylation genes downregulated in M1 condition. Gene coding for subunits of every components of the electron transport chain are represented. These genes tend to be upregulated in M2a 12–24 hours treatments.

**Fig 6:**
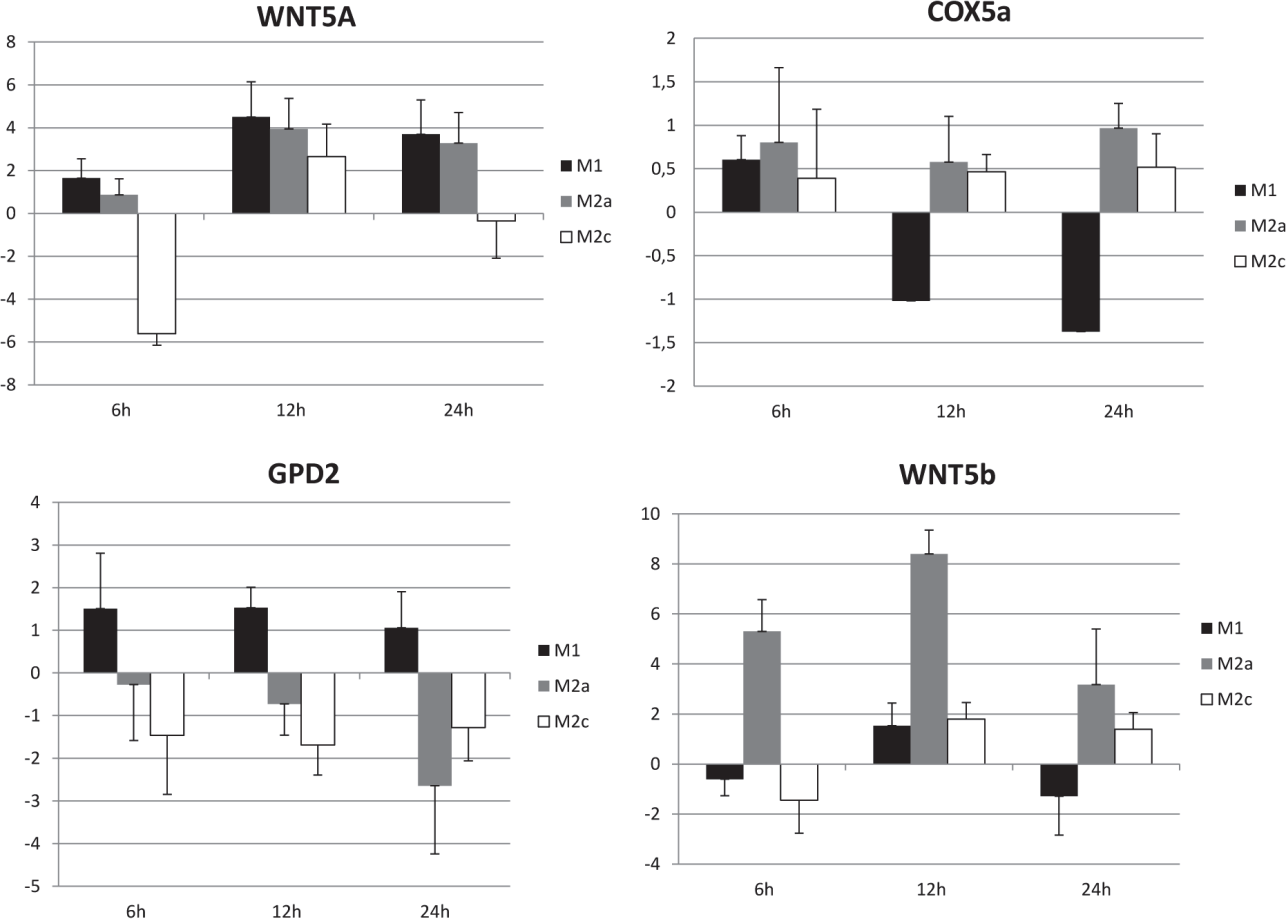
Validation of microarray data by quantitative pcr. mRNA expression level of *WNT5a*, *WNT5b*, *COX5a* and *GPD2* in RM, M1, M2a and M2c. axis: time course; Y axis: -ΔΔct.

GSEA was subsequently applied, focusing only on 12 and 24 hours data. At both time points there was the expected decline of expression in pathways and GO terms involved in inflammation and immune response. Gene sets involved in mitochondrial metabolism were significantly upregulated at 12 hours (Fig. 4, panel A). This network is composed mainly by genes involved in oxidative phosphorylation and respiratory electron transport (Fig. 4, panel B). Then, we assessed whether differences in enrichment significance at 12 and 24 hours were consistent with the gene expression patterns. There was a marked expression either in RM and M2a of genes mainly related to cellular respiration (ATP synthesis, Cytochrome c reductase, Cytochrome c oxidase and NADH-nicotinamide adenine dinucleotide hydride- dehydrogenases) (Fig. 5). In order to confirm these data, the expression level of some genes involved in these pathway was analyzed by qPCR (*COX5a* and *GPD2*, Fig. 6).

One of the most upregulated pathway, the “*Wnt signaling pathway”*, is composed by genes mainly involved in the non canonical Wnt pathway. Specifically, *WNT5b* was significantly overexpressed at each time point. In order to confirm these data, we also assessed by qPCR the expression of genes that compose the non canonical Wnt signaling pathway, *WNT5a* and *WNT5b* (Fig. 6).

## Discussion

In this study, we assessed time course and main features of gene expression during *in vitro* human Mϕ activation towards M1, M2a and M2c phenotypes.

We compared gene expression of each Mϕ phenotype to unstimulated resting Mϕ at the same time points, i.e. to *bona fide* time controls. In previous papers, subtypes of activated Mϕ were compared to each other, thereby highlighting differences in gene expression between subtypes [6, 17, 18]. Our experimental design can gauge the gene expression pattern which is specific of each subtype *per se*.

It should be noted that IL-10, used to trigger M2c deactivation, resulted in a gene expression pattern which, under our experimental conditions, although distinguishable from the time control of unstimulated Mϕ at 12 and 24 hours (Fig. 1), yet had no gene significantly modulated under our rather stringent bonds to recognize statistical significance (Table 1). In line with our results, previous papers found either no differentially expressed genes [31] or a difference in expression of a small number of genes [32; 33] in M2c. Conversely, M1 and M2a had a fair number of genes significantly up- and down-regulated (Table 1) as expected, among which the differential expression of *IRF-4* and -*5* between M1 and M2a is a remarkable example of positive control [34, 35].

Our first finding was that both M1 and M2a activations exhibited a detectable dynamic pattern within the time frame of our sampling. However, classical activation was quicker to ensue than alternative activation (Fig. 1), the latter being slower by at least 6 hours in reaching a fully differentiated gene expression pattern when compared to resting Mϕ. Thus, the clocks of the genetic programmes of M1 and M2a are not synchronous, and, with regard to the role of immunosuppressor cells played by M2a, the precession of M1 *vs* M2a activation in any case may leave a time window of several hours in which M1 biologic action goes on unopposed. To the extent that our *in vitro* data can be extrapolated to the *in vivo* setting, this time window of M1 action free from M2a influence is intrinsic to the Mϕ and independent of the environmental conditions, which may only widen it.

GSEA analysis showed a significant overlap of our microarray results (FDR<10^–4^ at all time points) of M1 and M2a activation with the data by Martinez et al. [6] (S2 Fig. for representative GSEA plots at 12 hours). However, our data also extended the list [6, 17, 18] of pro-inflammatory genes and genes involved in adhesion and phagocytosis which are upregulated in M1 Mϕ (S1 Fig.), thereby potentially providing ground to generate novel hypotheses on Mϕ biology and pathology.

Furthermore, GSEA analysis of M1 activation pointed out a fall in the expression of the genes of mitochondrial metabolism and cellular respiration (Fig. 2, panel A and B). The opposite pattern was detected in M2a activation (Fig. 4). These two patterns are consistent with the transcriptomic changes associated to insulin resistance and insulin sensitivity, respectively, in human adipocytes [36].

A remarkable detail of this insulin-resistant/insulin-sensitive pattern expressed by M1 and M2a, respectively, is the behavior of *GPD2*, a key enzyme in electron transport chain. Its significant upregulation in M1 and downregulation in M2a (Fig. 6) is consistent with its being a major target of metformin, which, by inhibiting *GPD2*, leads to a positive modulation of the cytosolic and mitochondrial redox state, sensitizes the hepatocyte to insulin action and decreases endogenous glucose production [37].

It should be re-emphasized that our findings were not the result of comparisons between M1 and M2a, but changes from the time control of resting Mϕ. Thus, from a purely metabolic viewpoint, M1 looks akin to a prototype insulin-resistant, M2a to a prototype insulin-sensitive cell. Accordingly, previous studies showed that alternative activated Mϕ are present in adipose tissue of lean insulin-sensitive subjects, whereas classically activated Mϕ are abundant in obese and insulin resistant subjects [38; 39]. Specifically, IL-4, secreted from Treg and eosinophils in adipose tissue of lean subjects, was shown to sustain alternative Mϕ activation. Moreover, PPARγ agonists, known to play insulin-sensitizing effects, were able to prime alternative activation [40]. Conversely, pro-inflammatory cytokines and adipokines secreted from adipocytes of obese subjects promote NFkB activation and inhibit insulin signaling [41]. Stated otherwise, from a purely metabolic standpoint, LPS and IFN-γ trigger an insulin-resistant [42, 43], IL-4 an insulin-sensitive [44] genetic programme in Mϕ. These findings are quite consistent with and supportive of the metaflammation hypothesis [45]. On these grounds, it might be speculated that the gene expression pattern of polarized Mϕ should be indicative of, and provide insight into, the status of insulin action *in vivo*.

M1 activation displayed a significant upregulation of the IL-10 signaling pathway, i.e. one major target of the deactivating action of M2a on M1 via release of IL-10 in the extracellular space [46]. Thus, the very same inducers of M1 activation also may sensitize the Mϕ to the deactivating action of IL-10, thereby setting the scenario to enhance the subsequent action of M2a. Analogous interpretation can be offered for the upregulation of *NFKBIA* and *SOCS3* during M1 activation.

STEM analysis showed that in M1 activation, the expression of cell migration and adhesion pathways displayed an early peak and a subsequent decline, and apoptotic pathways a linear progressive increase which did not plateau within the 24 hours of our experiment. Thus, in analogy to the timing of M1 and M2a activation, these data show that the clocks of adhesion and apoptosis in M1 are not synchronous, and it is an intrinsic property of the activated M1 to first adhere to a surface and only at a later time to fully activate the apoptosis program, independently of external cues. This makes the activated M1 to be well equipped, for instance, to undergo first rolling on and adhesion to activated endothelium and, only at a later time, to activate apoptosis well after it is resident within the vascular wall [47]. Moreover, inflammation and cell migration pathways, the expression of which decreases from 6 to 24 hours (Fig. 3), possibly reflect a fading acute response of Mϕ to pro-inflammatory cues.

M2a activation was characterized by no more than 50 differentially modulated genes (Table 1), when compared to resting Mϕ. A novel finding was that M2a also upregulated genes and gene groups at odds with their recognized anti-inflammatory role. Specifically, the Wnt pathways were upregulated in M2a, in particular *WNT5a* and *WNT5b* (Fig. 6), and both are players in the noncanonical Wnt signaling pathway. Wnt proteins, through the canonical and non canonical transduction pathways are involved in inflammatory processes [48] such as atherosclerosis, as well as in regulating cell proliferation and differentiation [49] and in cancer development [50].

In obese mice, *WNT5a* induced adipose tissue and Mϕ inflammation by JNK1(c-Jun N-terminal kinases-1) activation [51]. Recently, *WNT5a* was found to be expressed in adipose tissue Mϕ both in obese and obese type 2 diabetic subjects, in whom it putatively promotes low-grade inflammation [52]. Also *WNT5b* seems to be involved in the pathogenesis of type 2 diabetes [53], even if this is not fully elucidated. Consistent with our data, the canonical Wnt pathway recently was suggested to be a possible link between chronic inflammation associated with obesity and diabetic states and cancer development [54].

Previous studies reported an involvement of the canonical Wnt pathway in Mϕ polarization [55–57]. Our results expand those findings, suggesting that different Wnt pathways may be activated in the process of Mϕ polarization and that M2a might display a multifaceted functional profile in low grade inflammatory conditions, such as type 2 diabetes and atherosclerosis.

We acknowledge some limitations of our research. First, it is primarily a hypothesis generating, rather than a hypothesis testing, study. Even so, gene expression data are not supported by proteomic analysis. As a result, putative underlying molecular mechanisms, relationships and interactions remain to be investigated. In addition, owing to full anonymization of blood samples, donors’ biometric and clinical information, which could have been informative for data interpretation, were not available.

In conclusion, our study provided complete transcriptomic signatures of M1, M2a and M2c Mϕ subtypes. Several novel aspects regarding the time sequence and insulin-resistant/sensitive-like patterns of gene expression and the potential multifunctional role of M2a in low-grade inflammatory and apoptotic conditions were unveiled, resulting into novel hypotheses which need be tested in mechanistic studies.

## Supporting Information

S1 Table Significant genes in M1 Mϕ. Gene expression value of significant genes by Moderated t-test, BH correction p-value <0.05, in M1 in the three points of the time course (6, 12, and 24 hours) normalized to RM, M1, classical activated Mϕ.
(XLSX)

S2 Table Significant genes in M2a Mϕ. Gene expression value of significant genes by Moderated t-test, BH correction p-value<0.05, in M2a in the three points of the time course (12 and 24 hours) normalized to RM. M2a, classical activated Mϕ.
(XLSX)

S1 Fig Validation of microarray data by quantitative pcr. Panel A: validation of M1 marker genes *PTGS2*, *IL8*, *SOCS1*, *IL6*, *SOD2*; Panel B: validation of M2 marker genes: *CD200R*, *CD206*, *CD163*, *ALOX15*. X axis: time course hours; Y axis: -ΔΔct.
(TIF)

S2 Fig Experiment comparison with GSEA. Data published by Martinez and colleagues were retrieved from Gene Expression Omnibus (GSE5099) and compared with the present transcription profiles. Differentially expressed genes between “Macrophage at 7 days” condition and either “classical or M1 activated macrophages” or “Alternative or M2 activated macrophages” conditions were identified using the GEO2R tool, and the top 200 most upregulated or downregulated genes were kept to generate the M1_UP, M1_DN, M2a_UP, and M2a_DN gene sets. UP: upregulated genes; DN: downregulated genes.
(TIF)
