# Supplementary figures and images for: Transcriptomic Analysis of Human Polarized Macrophages: More than One Role of Alternative Activation?
Source: PLoS One. 2015 Mar 23;10(3):e0119751. doi: 10.1371/journal.pone.0119751 (PMC4370704; doi:10.1371/journal.pone.0119751)

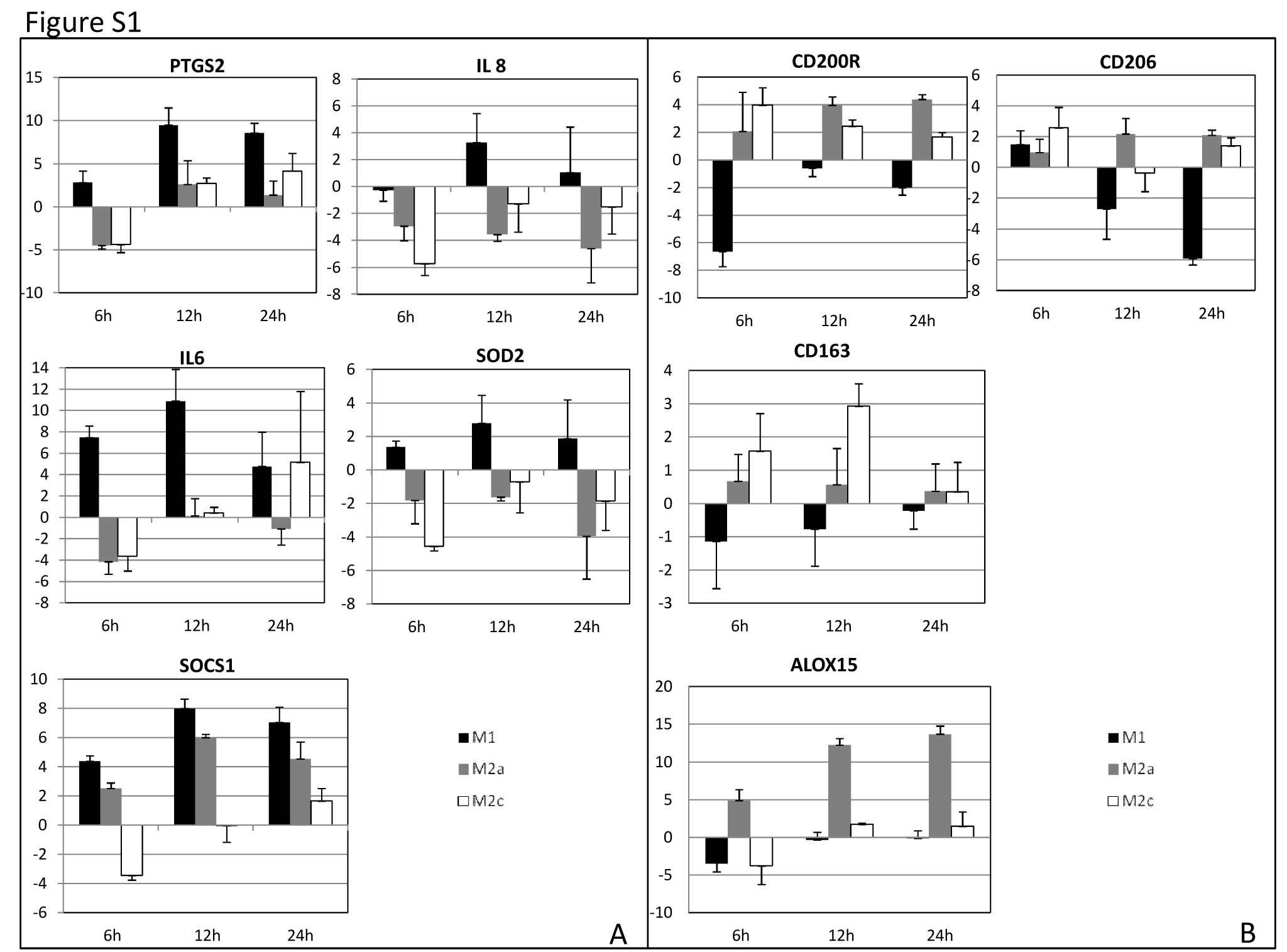

Supplement: S1 Fig — Panel A: validation of M1 marker genes PTGS2, IL8, SOCS1, IL6, SOD2; Panel B: validation of M2 marker genes: CD200R, CD206, CD163, ALOX15. X axis: time course hours; Y axis: -ΔΔct. (TIF) [file pone.0119751.s003.tif]

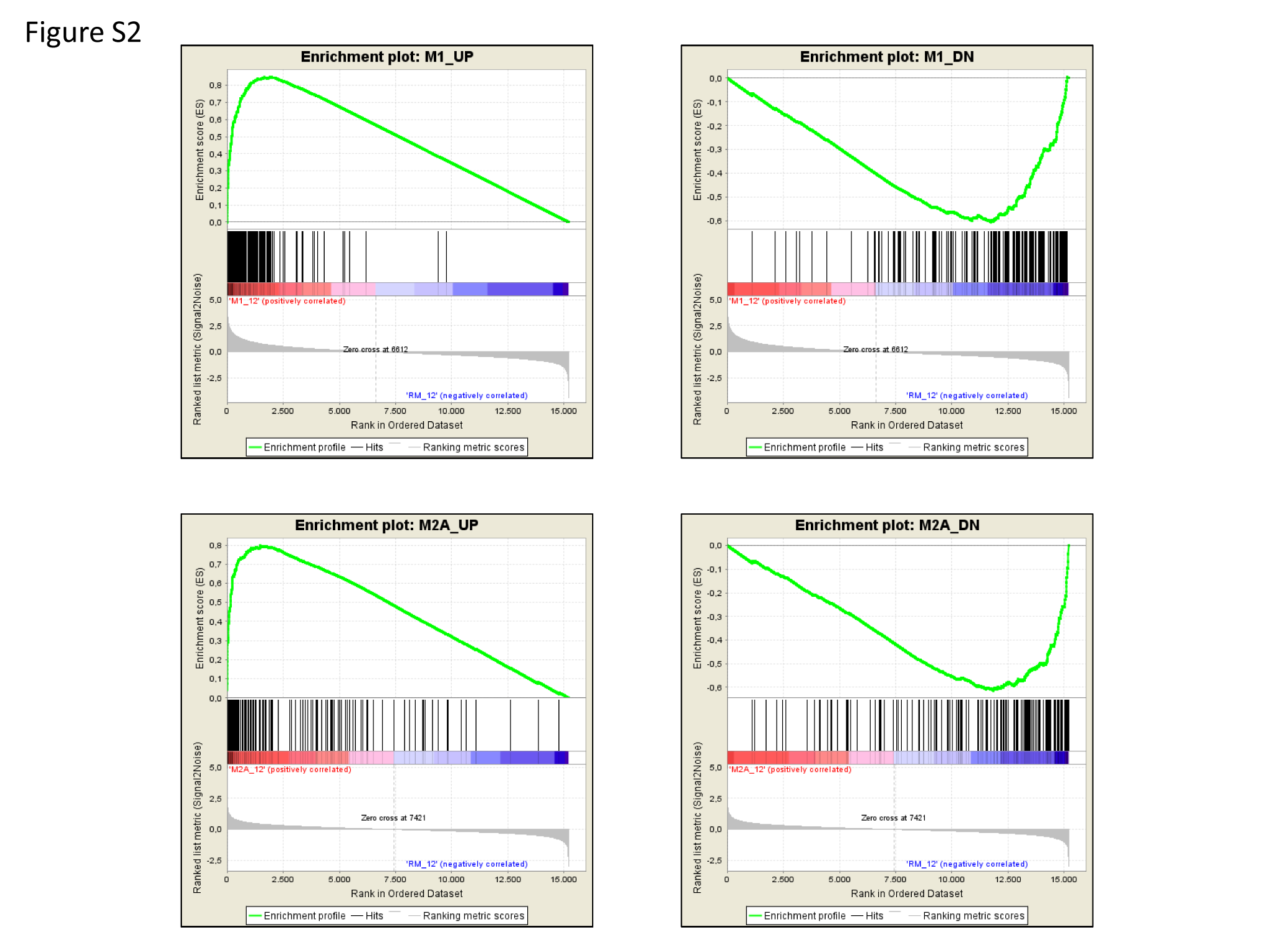

Supplement: S2 Fig — Data published by Martinez and colleagues were retrieved from Gene Expression Omnibus (GSE5099) and compared with the present transcription profiles. Differentially expressed genes between “Macrophage at 7 days” condition and either “classical or M1 activated macrophages” or “Alternative or M2 activated macrophages” conditions were identified using the GEO2R tool, and the top 200 most upregulated or downregulated genes were kept to generate the M1_UP, M1_DN, M2a_UP, and M2a_DN gene sets. UP: upregulated genes; DN: downregulated genes. (TIF) [file pone.0119751.s004.tif]
